# Antigen Specific Regulatory T Cells in Kidney Transplantation and Other Tolerance Settings

**DOI:** 10.3389/fimmu.2021.717594

**Published:** 2021-08-26

**Authors:** Min Hu, Natasha M. Rogers, Jennifer Li, Geoff Y. Zhang, Yuan Min Wang, Karli Shaw, Philip J. O’Connell, Stephen I. Alexander

**Affiliations:** ^1^Centre for Transplant and Renal Research, Westmead Institute for Medical Research, Sydney, NSW, Australia; ^2^Faculty of Medicine and Health, The University of Sydney, Sydney, NSW, Australia; ^3^Centre for Kidney Research, Children’s Hospital at Westmead, Sydney, NSW, Australia

**Keywords:** kidney, transplantation, Treg, antigen, tolerance

## Abstract

Kidney transplantation is the most common solid organ transplant and the best current therapy for end-stage kidney failure. However, with standard immunosuppression, most transplants develop chronic dysfunction or fail, much of which is due to chronic immune injury. Tregs are a subset of T cells involved in limiting immune activation and preventing autoimmune disease. These cells offer the potential to provide tolerance or to allow reduction in immunosuppression in kidney transplants. The importance of Tregs in kidney transplantation has been shown in a number of seminal mouse and animal studies, including those with T cell receptors (TCRs) transgenic Tregs (TCR-Tregs) or Chimeric Antigen Receptor (CAR) Tregs (CAR-Tregs) showing that specificity increases the potency of Treg function. Here we outline the animal and human studies and clinical trials directed at using Tregs in kidney transplantation and other tolerance settings and the various modifications to enhance allo-specific Treg function *in vivo* and *in vitro*.

## Introduction

Kidney transplantation is currently the preferred treatment for patients with end-stage kidney disease (ESKD). The primary limitation of long-term kidney allograft survival has been chronic immune-mediated rejection. This is due to both cellular and humoral pathways as well as innate immune factors and possibly other pathways. Major improvements have occurred over time with improved and more specific immune suppression particularly targeting T effector cells. However, because of the deleterious effects of immunosuppression a major goal has been to achieve immune tolerance to the transplant.

In organ transplantation, there has been a longstanding interest in transferring therapies that create tolerance to the clinic (1). In kidney transplantation, a number of bone marrow-based strategies creating temporary or permanent donor hematopoietic chimerism have reached clinical trials. The first of these was done at Massachusetts General Hospital (MGH), followed closely by Stanford, John Hopkins and North Western (2–6).

Regulatory T cells (Tregs) are a subset of T cells that suppress immune activation and limit autoimmunity in the periphery (7). A number of cell types with immune-regulatory function have been characterized as Tregs, though the one best understood, at present, are the CD4^+^FOXP3^+^ Tregs. These either arise in the thymus (tTregs) or develop from peripheral CD4^+^ T cells that convert into CD4^+^FOXP3^+^ Tregs as peripherally derived Tregs (pTregs) (8). Previously, it had been thought that peripheral tolerance due either tTregs or pTregs would be insufficient for long standing tolerance. However increasing understanding of Tregs and other regulatory subsets has led to the possibility that transplant tolerance allowing reduction or cessation of immunosuppression could be achieved using regulatory cell subsets (9).

We and others have shown a role for Tregs in 1) protecting against autoimmune kidney disease, 2) maintaining tolerant kidney grafts, 3) preventing human islet allograft and pig xenograft rejection in humanised mouse models, and 4) preventing kidney allograft loss in mouse models of kidney tolerance (10–13). In addition to our studies, there have been 3 bone marrow transplant (BMT) trials reporting the use of Tregs in graft *versus* host disease (GVHD), and there are current human trials using Tregs in autoimmune diabetes at UCSF (14–17). Clinically, polyclonal human Tregs have been shown to be safe to infuse in kidney transplant patients in the multi-centre One Study (18) and the safety of this study suggests that Tregs may be the next frontier of cell therapy (19–21). This has also been achieved at North Western University in kidney transplant recipients with *ex-vivo* expanded Tregs (22). This group has also developed protocols for *ex-vivo* expanded allo-antigen specific Tregs (23).

In this review, we focus primarily on antigen specific Tregs induction in kidney transplantation tolerance and other tolerance studies in animal models and human studies of kidney transplantation and discuss several approaches to generate and expand antigen specific Tregs which can protect against transplant rejection.

## Regulatory T Cells

Tregs that limit autoimmunity have been studied for a long time. Almost 20 years ago the identification of a CD4^+^CD25^+^ subset as necessary to protect against autoimmunity and limit alloresponse and the subsequent identification of Foxp3 as a master transcription factor identified this regulatory subset (24–27). Further, the separation of these FOXP3^+^ Tregs into tTregs or pTregs developed in the periphery *in vivo* has helped shape an understanding of their action (28). Tregs can function through multiple pathways to induce tolerance. Tregs are likely to have a number of functional subsets that mirror effector T cells (28). Naïve CD4^+^ T cells can be induced into FOXP3^+^ Tregs *in vitro* as iTreg in this review. tTregs with epigenetic changes, provide stability of demethylated FOXP3 especially at the Treg‐specific demethylated region (TSDR) site, making them are more resistant to conversion to other CD4 T cell phenotypes (29); pTregs and iTregs without TSDR demethylation, appear at risk of conversion into pathogenic subsets of CD4 T cells, including Th17 (30).Many subsets of effector Tregs express lineage-specific transcription factors in combination with the transcription factor B-lymphocyte-induced maturation protein 1 (BLIMP1) which acts as a repressor. The important role of IL-2 and IL-4 in driving alloantigen specific Tregs in transplantation has recently been demonstrated (31). It has also been shown that acquisition of antigen-specific memory by Tregs is associated with more potent function (32). Further recent data suggests that memory Tregs are more potent than naïve Tregs as shown in antigen-specific models of skin inflammation where response to self-antigen imprints regulatory memory in skin tissues and this has also been shown in rat cardiac transplant models (33). Of interest, antigen-specific CD4^+^ type 1 regulatory T cells that express IL-10 and/or TGF-β (Tr1) can be induced independently of foxp3 expression, can limit antigen-specific immune responses and may also be important as mediators of transplant tolerance (34, 35). Further identification of memory markers on effector Tregs in transplant settings suggests these may be more potent (36).

## Donor Antigen-Specific Tregs in Animal Transplant Models

**Figure 1:**
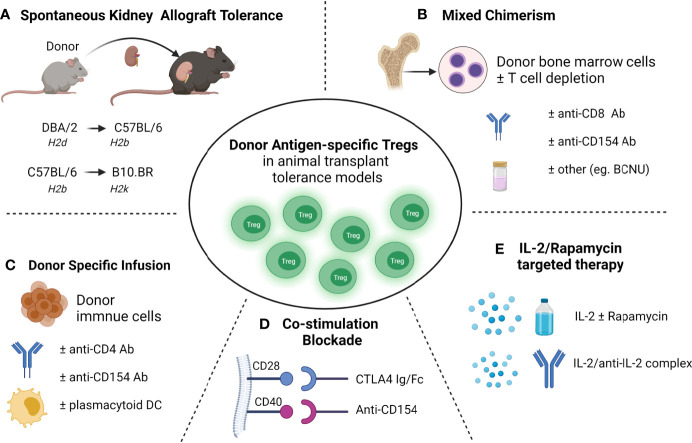
Donor antigen-specific Tregs developed in transplant tolerance mouse models **(A)** Spontaneous acceptance of kidney allotransplant tolerance. **(B)** Mixed chimerism induced allo-transplantation tolerance *via* bone marrow transplantation. **(C)** Donor-specific transfusions (DST) induced allotransplantation tolerance. **(D)** Blockade of the B7-CD28 (using CTLA-4 Ig/Fc) or/and CD40-CD40L (using anti-CD154 mAb) co-stimulatory pathway induced transplantation tolerance; **(E)** and IL-2 and rapamycin targeted therapies induced transplantation tolerance.

In transplantation, the key goal is to achieve donor-specific transplant tolerance in which the donor allograft is accepted without long-term immunosuppressive requirements, and the recipient remains capable of responding to pathogenic microorganisms and other alloantigens (37). Donor-specific tolerance of allografts arises spontaneously in various animal models (11, 38–40) and has been well-documented in various transplant populations (**Figure 1**), including our study in a pediatric liver transplant recipient with full hematopoietic chimerism (41). As well as Treg therapy, several approaches have been used to achieve transplant tolerance in animal models, including costimulatory blockade, hematopoietic donor chimerism using BMT and stem cell transplantation, targeting of effector cell activation and recruitment, and donor-specific transfusions (DST). Among these approaches, donor-antigen specific Foxp3^+^ Tregs have been found to play the crucial role in the maintenance of allograft tolerance in several transplant animal models.

### Donor Antigen-Specific Tregs in Spontaneous Acceptance of Kidney Allografts

Donor-specific tolerance of renal allografts occurring spontaneously across certain MHC mismatched mouse strains is a valuable model for studying transplant tolerance (11, 38–40). Studies showed spontaneous kidney allografts tolerance occurred in the DBA/2 (H2^d^) to C57BL/6 (H2^b^) transplant model and was associated with T cell mediated immune regulation requiring TGF-β (40). In a spontaneous kidney allograft tolerance mouse model with C57BL/6 mice as donors and B10.BR (H2^k^) as recipients, we found Foxp3^+^ Tregs increased in tolerant kidney allografts (39). Further we and others demonstrated that Foxp3^+^ Tregs were essential for tolerance induction in spontaneously accepted kidney allograft from DBA/2 to C57BL/6 mice, where depletion of CD4^+^CD25^+^ Tregs or Foxp3^+^ Tregs abolished kidney allograft tolerance (11, 42). Using DEREG mice (C57BL/6) that carry the diphtheria toxin receptor and enhanced green fluorescent protein (DTR-eGFP) transgene under control of the Foxp3 promoter, we identified donor -antigen-specific Foxp3^+^ Tregs that were developed in DBA/2 kidney allografts in C57BL/6 recipient mice and confirmed dominant and donor antigen specificity of kidney allograft Tregs *in vivo* in Rag-/- mice (11). These donor antigen-specific Foxp3^+^ Tregs expressed elevated levels of TGF-β, IL-10, interferon gamma (IFN-γ), BLIMP1 and the chemokine receptor 3 (Cxcr3) (11). These studies demonstrated the crucial role of Foxp3^+^ Tregs in transplant tolerance and the therapeutic potential of donor antigen-specific Tregs in clinical settings to improve transplant outcomes.

### Donor Antigen-Specific Tregs in Allo-Transplantation With DST

In transplantation, Tregs have been shown to develop in the recipients after DST and the critical role of Tregs has been reported in DST models with mismatch at both major histocompatibility class (MHC) class I and class II (43–48). However, other studies reported equal expansion of recipient transplant-specific Tregs in the rejecting and tolerant heart allografts induced through DST combined with anti-CD154 mAb, and without Treg expansion in the spleen (49). Although DST combined with cyclophosphamide treatment failed to prolong BALB/c (H2^d^) islet allograft survival in C57BL/6 (H2^b^) host mice, additional infusion of either donor-reactive Tregs (5 × 10 ^6^) or polyclonal Tregs (25 × 10 ^6^) led to prolonged survival of allogeneic islets in > 70% of C57BL/6 host demonstrating the critical role of Tregs in DST for induction of tolerance (46). Other studies have also reported DST combined with anti-CD154 mAb and plasmacytoid dendritic cells (pDCs) induced BALB/c heart allograft tolerance in C57BL/6 hosts in which Tregs developed and expanded in the lymph node (47). In a MHC-mismatched skin allotransplant mouse model, mice pre-treated with anti-CD4 monoclonal antibody (mAb) and DST achieved dominant tolerance which was mediated by Tregs in a CTLA-4 and IL-10-dependent manner. However CD4^+^CD25^+^ Tregs from graft tolerant mice had no demonstrable specificity for the tolerizing donor antigens (50). In models using DST with additional immunological manipulation, expanded alloreactive Tregs occurred predominantly through the direct pathway of allorecognition (51) and long-term graft acceptance predominantly relied on indirectly activated Tregs (45, 52). Interestingly, in a single MHC-I mismatch skin transplant model, we demonstrated dominant F1 skin-allograft [C57BL/6.C(H-2^bm1^) × C57BL/6 (H2^b^)] tolerance induced by a single DST across an unmanipulated C57BL/6 host (53). In this model, expansion of antigen-specific Foxp3^+^ Tregs in F1 grafts and spleens of recipient mice were due to thymus-derived Foxp3^+^ Treg proliferation that were indirectly activated. The essential role of Foxp3^+^ Tregs in this model was confirmed by depletion of Foxp3^+^ Tregs in DEREG mice which abrogated F1 skin graft tolerance (53).

### Donor Antigen-Specific Tolerance in Allo-Transplantation Combined With BMT

Kidney transplant tolerance in a clinical setting has been achieved with non-myeloablative BMT to induce hematopoietic mixed chimerism, but is limited by the risk of GVHD, and loss of both mixed chimerism and tolerance (3, 54, 55). Mixed chimerism achieves transplant tolerance through both central and peripheral depletion of alloreactive cells (54). More recent data support an important role for Tregs in mixed chimerism, both in limiting GVHD and maintaining chimerism with host Treg cells involved in preventing chronic allograft rejection and donor Treg cells controlling GVHD in animal models and transplant recipients (56–61). Mouse studies have shown an important role for host Treg cells in tolerance using nonmyeloablative BMT, costimulatory blockade and total body irradiation to achieve mixed chimerism across a full MHC mismatch (58). Mixed chimerism only occurred in the presence of host Tregs in host mice and allogeneic T cell-depleted BMT in a non-lymphoablative irradiation regimen (57). Moreover, host Tregs specific for directly presented donor antigens prevented acute rejection only of skin and heart allografts, while host Tregs specific for both directly and indirectly presented alloantigens prevented both acute and chronic rejection in the same models (57). Using a non irradiation regimen in which the host received, anti-CD154 mAb, a single dose of depleting CD8 mAb, N,N-bis(2-chloroethyl)-nitrosourea (BCNU, an alkylating agent), and allogeneic BMT from methyl-guanine-methyl-transferase (MGMT) transgenic mice, high levels of donor mixed chimerism without GVHD were achieved across fully MHC mismatched mice leading to donor-specific skin allograft tolerance (56). MGMT is a DNA repair enzyme that removes BCNU from guanine and limits BCNU toxicity, enhancing engraftment of donor hematopoietic cells. Importantly, both donor and host Foxp3^+^ Tregs were expanded in this model, suggesting their important role for the maintenance of tolerance in mixed chimerism (56). In non-human primates (NHPs) where kidney allograft tolerance was achieved with the development of transient mixed chimerism using T cell-depleted BMT and costimulatory blockade without myeloablation, Foxp3^+^ Tregs were found to be enhanced in tolerant recipients (62). Thus, mixed chimerism and Tregs are likely to be synergistic in achieving transplant tolerance and limiting GVHD in clinical transplantation (63–65).

### Donor Antigen-Specific Tregs in Costimulation Blockade–Induced Transplant Tolerance

In several transplant model systems, blockade of the B7-CD28 (using CTLA-4 Ig/Fc) or CD40-CD40L (using anti-CD154 mAb) co-stimulatory pathway induced donor graft tolerance (66–72). The impact of Treg numbers and functionality on the efficacy of costimulation blockade, and the requirement of Tregs in transplant induction *via* co-stimulation blockade is an area of active investigation (67, 68, 73, 74). Although CTLA-4–Ig treatment in C57BL/6 naïve mice causes a decrease in Treg and Helios^+^Foxp3^+^ Tregs, it prolongs fully mismatched BALB/c heart allograft survival in C57BL/6 recipients. However, it does not protect single MHC-II mismatched heart allografts in C57BL/6 recipients in which long-term allograft survival was Tregs dependent (73). In a different model, treatment with CTLA4-Ig abolished skin allograft tolerance induced by IL-2/anti–IL-2 complex suggesting that allo-Treg development requires costimulation (75). Others reported that in the presence of B7:CD28 and CD40:CD40L co-stimulatory blockade, the suppressive function of CD4^+^CD25^+^ Tregs was activated, suppressing the proliferation of CD4^+^ effector cells (67). Experiments *in vitro* by Vogel et al. found that co-stimulatory blockade primed Foxp3^+^ Tregs to be more suppressive than naïve Foxp3^+^ Tregs (76). Depletion of CD4^+^CD25^+^ Tregs in recipient mice abolished heart allograft tolerance induced by CD154 mAb treatment (77). In an islet xenotransplant pig-mouse model, we found expanded Foxp3^+^ Tregs populations in the xenograft, spleen and draining lymph nodes that were induced by treatment with CTLA-4 Fc and anti-CD154 mAb. Once again activated Foxp3^+^ Tregs had more potent regulatory function *in vivo* than naive Tregs (68). Further, depletion of Foxp3^+^ Tregs at induction (unpublished) or maintenance (68) in recipient mice with the blockade of B7-CD28 or CD40-CD40L abolished transplant tolerance thereby confirming the essential role of these cells. However, antigen specificity of Treg after the induction of tolerance does not appear to be universal. Using a combination of nondepleting CD4, CD8, and CD154 mAb to induce fully mismatched C57BL/10 (B10, H-2^b^) skin allograft tolerance in CBA (H2^k^) mice, antigen specific tolerance to the second skin allograft was shown to be mediated by CD4^+^CD25^+^ Tregs; but these Tregs from tolerant mice had no demonstrable donor antigen specificity after adoptive transfer (78).

### Donor Antigen-Specific Tregs in IL-2 and Rapamycin Targeted Therapies

The cytokine interleukin-2 (IL2) is a key regulator of immune cells including Tregs that constitutively express the high‐affinity IL‐2Rα (CD25) and are highly sensitive to very low doses of IL‐2 (79–81). In the clinic, low-dose IL-2 therapy has been shown to selectively expand FOXP3 Tregs and used successfully to treat chronic GVHD (82), hepatitis C virus–induced vasculitis (83), systemic lupus erythematosus (SLE) (84), and type 1 diabetes (85, 86). In a humanized mouse model, we demonstrated that the infusion of *in vitro*–expanded human FOXP3^+^ Tregs in the presence of IL-2 and rapamycin prevented porcine neonatal islet cell cluster xenograft rejection (87). Low dose IL-2 treatment in the host mice led to expansion of Foxp3^+^ Tregs *in vivo* and prolonged allograft survival in a full MHC-mismatch corneal transplant model of C57BL/6 grafts to BALB/c hosts, and these *in vivo* expanded Tregs also demonstrated donor antigen specificity (88). Further, IL-2 treatment of host mice preferentially expanded infused donor antigen-specific Tregs generated *in vitro*, and improved the skin allograft survival in a single MHC-mismatch mouse model (89). Using IL-2/anti-IL-2 antibody complexes has also been shown to induce Foxp3^+^ Tregs expansion, prolong islet allograft survival (90) and protect against proteinuric chronic kidney disease (91). In our study, low dose of IL-2 treatment alone failed to prolong islet allograft survival in humanized mice despite expandingFOXP3^+^ Tregs *in vivo* (92). Graft survival depended on the balance of effector T cells and Tregs. Rapamycin has been shown to be pro-tolerogenic by allowing expansion of human and mouse Tregs while also suppressing CD4^+^ T effector cells in experimental studies (93–96). Whilst low-dose rapamycin treatment did not lead to either Treg expansion *in vivo* or prolongation of islet graft survival in humanized mice, the combination of IL-2 and rapamycin led to an expansion of Tregs and prolonged human islet allograft survival in this humanized mice model (92). The combination of IL-2 and rapamycin has also been reported to prolong skin graft survival in minor antigen mismatch and semi-allogeneic mouse recipients (97). These studies suggest a combination of agents that selectively expand Tregs *in vivo* whilst limiting effector T-cell expansion will be required to protect graft rejection if these therapies are to be translated in the clinic. Although these results in rodent studies are encouraging, more work needs to be done to ensure that T cell activation *in vivo* is limited to the Treg population only. IL-2, at the appropriate dose, can activate effector T cells, and a clinical trial of low dose IL-2 in liver transplant recipients had to be ceased due to increased rejection without evidence of Treg induced suppression (NCT02949492).

## Potential Methods to Generate and Induce Allospecific Tregs *Ex Vivo*

**Figure 2:**
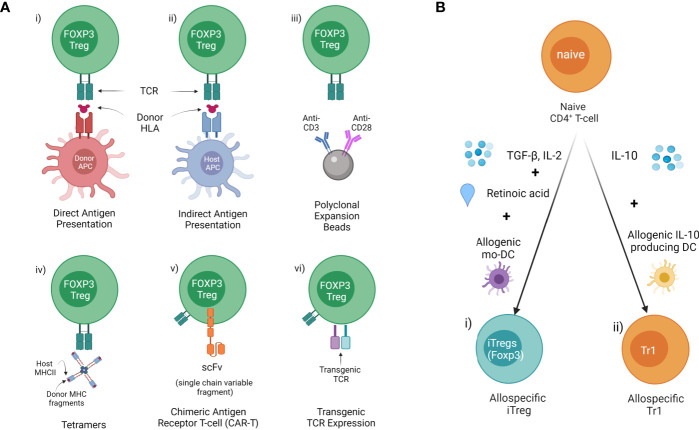
Methods of allospecific Treg derivation **(A)** Generating donor antigen-specific Tregs from FOXP3^+^ Tregs (CD4^+^CD25^+^CD127^-^) precursors. This includes donor HLA presented to generate i) direct antigen expanded (donor APC/MHCII) or ii) indirect antigen expanded Tregs ed (host APC/MHCII). iii) Polyclonal expansion with antiCD3/CD28 beads. iv) Isolation of specific Tregs using Tetramers composed of host class II MHC & donor MHC fragments. v) Insertion of antibody fragment scFv connected to TCR to create a CAR. vi) Genetic modification of Tregs with transgenic TCR. **(B)** Inducing donor antigen-specific i) iTreg and ii) Tr1 from naïve CD4^+^ T cells.

Concerns around non-specific immune suppression and the need to increase potency has led to the development of antigen-specific Tregs which display more efficient suppressive function than naïve Tregs. There are several methods to generate donor antigen-specific Tregs from FOXP3^+^ Tregs (CD4^+^CD25^+^CD127^-^) precursors, and antigen-specific iTregs or Tr1 from naïve CD4^+^ T cells for therapy in transplantation (**Figure 2**) (98, 99).

### Direct Antigen Expansion With Donor HLA Expressed by APC

Human Tregs expanded in the presence of donor antigen *ex vivo* demonstrate enhanced suppressive function and were more effective for protecting skin allograft rejection in a humanized mouse model (100). Our group has identified xenospecific human CD27^+^HLADR^+^ Tregs generated in the presence of pig donor peripheral blood mononuclear cells (PBMCs) in a mixed lymphocyte reactions (MLR) system which demonstrated more effective protection of pig islet xenograft rejection in a humanized mouse model (unpublished). Allospecific Tregs were generated using isolated CD4^+^CD25^+^CD127^-^ Tregs from PBMCs of healthy volunteers in the presence of soluble 4-trimer CD40 ligand (CD40L)-activated donor B cells (23) or skin-derived myeloid DCs (101). Further studies have shown that human allospecific Tregs can be generated in a MLR system from PBMC of uremic patients in the presence of donor PBMCs under conditions of costimulatory blockade with belatacept (102). Allospecific Tregs were generated in different MLR system in which sorted CD4^+^CD25^+^CD127^-^ Tregs from transplant recipient’ PMBCs were expanded with donor B cells activated using K562 cells and expressing human CD40L (100). These allospecific Tregs maintained high levels of FOXP3 expression and TSDR (100, 102), and demonstrated safety for cell therapy in clinical kidney transplant trials (18, 100, 102).

### Indirect Antigen Expansion With Donor HLA Fragments Presented by Host APC/MHC

Another approach has been the use of indirectly presented HLA molecules in the context of recipient MHC using antigen presenting cells (dendritic cells or B cells) pulsed with donor antigen. This may potentially be more effective for suppression of chronic rejection (103). However, this is often limited by the small clone size of TCR recognizing the peptide in the context of self-MHC or the development of inhibitory constimulatory molecules by the APC.

### Isolation of Specific Tregs Using Tetramers Composed of Host Class II MHC & Donor MHC Fragments

This has the advantage of having a broad range of T cell receptors (TCRs) in the selected population. It still requires Treg expansion and sorting. It also has issues around the initial selection of Tregs and it may potentially be useful in displaying a range of targets. However MHC Class II tetramers have proved less efficient than Class I and may be a barrier to larger scale clinical use (104).

### Insertion of Allospecific TCR

The use of TCRs against an alloantigen was first used by Simpson and her group using TCR transgenic mice against the H-Y antigen (105, 106). Here Tregs directed against H-Y could induce tolerance against a minor antigen (107, 108). TCR-transduced Tregs specific for allogeneic MHC class II molecules induced long-term survival of partially MHC-mismatched heart grafts when combined with short-term adjunctive immunosuppression (109). This demonstrated the capacity and increased potency of TCR targeted Treg therapy. More recently this approach has been used in cancer against minor H-A antigens but may have a future role in transplantation tolerance (110).

### Car-Tregs

Antibody fragment scFv connected to the intracytoplasmic tail of the TCR and associated signaling molecules are transduced into Tregs to create a chimeric antigen receptor (CAR) Treg (111). The targeting of Tregs in organ transplantation and in autoimmunity to the transplanted cells or autoimmune tissues is an attractive option. The potency of antigen targeting was first shown by the Simpson group as described above. T cell specificity has also been achieved using antibody fragments linked to intracellular TCR signalling domains called CARs. CAR T cells have been used most impressively in the treatment of CD19 lymphoma using CD8 T cells engineered with a scFv chain from an antibody against CD19 fused to the intracellular component of the T cell receptor (112–114). These hybrid receptors are comprised of a scFv derived from monoclonal antibodies or an antigen-binding fragment (Fab) fused to CD3-ζ transmembrane and end domains, the intracellular functional component of the TCR. This approach has been used to direct T cells against cancer antigens, most spectacularly against CD19. Further modifications enhance the survival and activity of the Treg and drive the functionality of the T cells have been undertaken (115).

The efficacy of CAR Tregs directed against alloantigens has been demonstrated using HLA-A2 specific CAR T cells *in vitro* and in transplant models (116–118). However, CAR Tregs while limiting skin graft rejection in naïve mice cannot prevent skin graft rejection in sensitized mice (119). Many strategies exist to improve the isolation and expansion of Tregs and subsequent genetic modifications to alter cytokine signalling, enhance regulatory elements or inhibit signals that supress regulation and develop pathways for testing in large animals and human trials (120).

### Generating Allospecific iTregs From Naïve CD4^+^ T Cells

iTregs are induced to express FOXP3 from naïve CD4^+^ under specific condition *in vitro*, but iTregs without TSDR demethylation, appear more susceptible to conversion into pathogenic subsets of CD4^+^ T cells (30). iTregs induced with TGF-β in the presence of all-trans retinoic acid (ATRA) and rapamycin demonstrated robust suppressive function *in vitro* but not *in vivo*, in the humanized GVHD mouse model (121). Interestingly, allospecific iTregs that were induced with TGF-β1, IL-2, and ATRA in the presence of allogeneic monocyte-derived dendritic cells, can specifically suppress donor allo-responses but not third-party allo-responses, and maintain suppressive function in the presence of pro-inflammatory cytokines, despite methylation of the FOXP3 TSDR (122).

### Generating Allospecific Tr1

Tr1 cell therapy in GVHD trials including NCT03198234 suggested therapeutic possibility in the treatment of transplantation rejection (123). Allospecific Tr1 were enriched in cultured CD4^+^ T cells that were stimulated with allogeneic IL-10-producing DCs generated from CD14^+^ monocytes in the presence of IL-10 (124). These allospecific Tr1 showed specific suppression function to donor alloresponse *in vitro* and maintained a tolerogenic gene expression profile *in vivo* (124).

## Antigen-Specific Tregs in Transplant Clinical TRIALS

Given the central role of T-cells in downstream effector function, Tregs have been studied as a potential therapeutic in human solid organ transplantation. Sixteen Treg clinical trials that have focused on feasibility, safety and preliminary efficacy of infused Tregs to reduce the dose of calcineurin inhibitor (CNI)-based immunosuppression have been reported. However, the majority of reported clinical trials using Tregs lack the capacity to robustly identify mechanistic effects and much of our understanding of underlying mechanisms relies on extrapolation of results from rodent and non-human primate studies.

**Figure 3:**
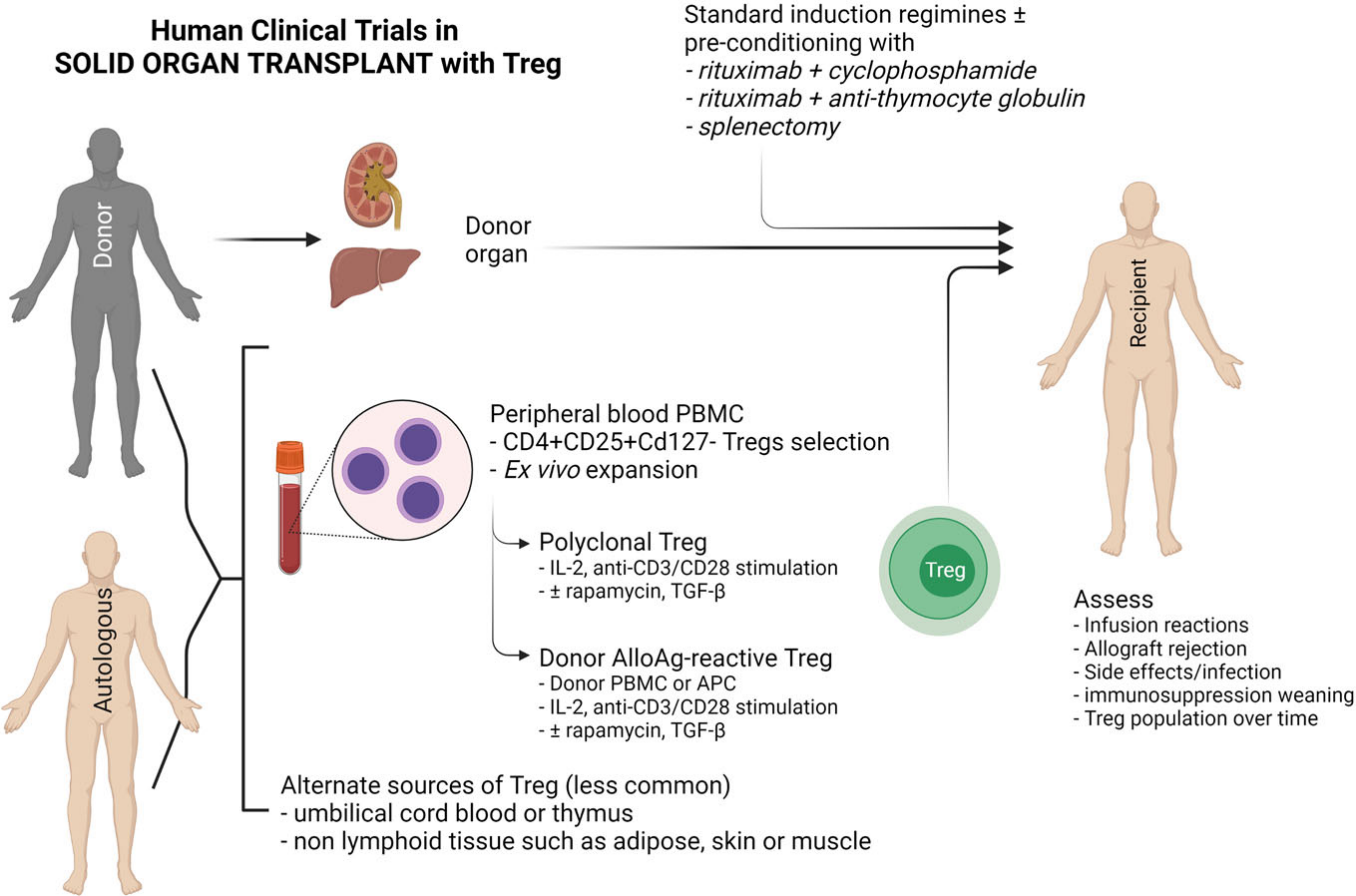
Schematic diagram of current clinical Treg trials in kidney and liver transplantation. This diagram indicates the sources of FOXP3^+^ Tregs (CD4^+^CD25^+^CD127^-^), the two main methods of Treg expansion and assessment in clinical trials.

Autologous or donor-derived peripheral blood is the most common source of Tregs used in clinical trials, although alternate sources such as umbilical cord blood and paediatric thymus, non-lymphoid tissue such as skin, adipose tissue and muscle have been explored (125) (**Figure 3**). Peripheral blood contains only a small proportion of Tregs that require purification *via* CD25^+^ cell selection, with or without CD8^+^ depletion. *Ex vivo* expansion of these naturally-occurring Tregs can be achieved through various means, but the choices are dependent on whether polyclonal or donor alloAg-reactive Treg (darTreg) are required (100). darTregs are theoretically more potent, however the majority of trials have expanded polyclonal Tregs with a combination of IL-2 and anti-CD3/CD28 magnetic bead stimulation ± rapamycin or TGF-β, although this process may require a greater cell infusion dose to achieve donor-specific suppressive effects (126–129) (**Figure 3**).

Clinical studies in kidney or liver transplantation have demonstrated Treg safety in early phase I/II studies. The One study (18), which included polyclonal Treg (n = 12 and 11 at Guy’s and Oxford University Hospitals, UK and Charité Hospital, Germany) and darTregs (n = 2) at the University of California San Francisco Medical Centre and (n = 3) at Massachusetts General Hospital, USA, did not demonstrate significant adverse events such as rejection. However, infusion of darTregs following a preconditioning regimen of splenectomy, rituximab with cyclophosphamide, or rituximab with rabbit anti-thymocyte globulin in living donor kidney transplantation resulted in significant rejection risk following immunosuppressive drug weaning (130). This highlights the caution required to achieve *in vivo* translation of cell therapies – despite demonstrating functionally suppressive Tregs *in vitro*, there was either insufficient Treg dosage, loss of regulatory function *in vivo*, or significant depletion of recipient Tregs. Additional studies of darTregs in liver transplantation (deLTa, NCT02188719) was not completed within the study timeframe, and several additional studies (ThRIL, NCT02166177; ARTEMIS NCT02474199) are either not formally reported or yet to commence recruiting (LITTMUS, NCT03654040). Tr1 cell therapy in two kidney transplant patients in the One Study group showed Tr1 cells expand and Tr1-cell tolerogenic gene expression profile remains stable even under active immunosuppressive treatment (124).

## Conclusions

The safety of infusing mildly manipulated Tregs has been demonstrated in clinical trials of kidney transplantation. Promising animal studies suggest that specific Tregs or modified Tregs may be more potent and allow further reduction in immunosuppression or true tolerance. There are caveats with CAR T cells failing to suppress rejection in sensitized mice and primate studies demonstrating Treg conversion. However overall, the accumulating evidence suggests that these barriers can be overcome and allo-specific Tregs therapy translated into the clinical practice.

## Author Contributions

MH drafted the sections of donor antigen specific Tregs in animal transplant models, revised and edited the paper. NR drafted the section of antigen specific Tregs in transplantation clinical trials, revised and edited the paper. JL drafted the figure and edited the the paper. GZ, YW, and KS revised the draft. PO’C revised the draft and edited the paper. SA drafted the sections of introduction, potential methods to induce allospecific Tregs *ex vivo*, and conclusions, edited and finalized the paper. All authors contributed to the article and approved the submitted version.

## Funding

This work was supported by the National Health and Medical Research Council of Australia (NHMRC) grants, JDRF/Australian Research Council, and University of Sydney. MH was awarded an early career fellowship from the NHMRC (GNT1013185), a Deputy Vice Chancellor University of Sydney research fellowship (IRMA178768). NR is a recipient of an NHMRC career development fellowship (GNT1158597). PO’C was a recipient of a senior practitioner fellowship from the NMHRC (GNT1125456) and the principal investigator of JDRF/Australian Research Council grant 4-SRA-2016-265-M-B. SA is a chief investigator A of NHMRC Ideas Grant GNT1183810.

## Conflict of Interest

The authors declare that the research was conducted in the absence of any commercial or financial relationships that could be construed as a potential conflict of interest.

## Publisher’s Note

All claims expressed in this article are solely those of the authors and do not necessarily represent those of their affiliated organizations, or those of the publisher, the editors and the reviewers. Any product that may be evaluated in this article, or claim that may be made by its manufacturer, is not guaranteed or endorsed by the publisher.
